# Insulin pump therapy with and without continuous glucose monitoring in pregnant women with type 1 diabetes: a prospective observational Orchestra Foundation study in Poland

**DOI:** 10.1007/s00592-022-02020-9

**Published:** 2023-01-19

**Authors:** Katarzyna Cypryk, Ewa Wender-Ozegowska, Katarzyna Cyganek, Jacek Sieradzki, Kinga Skoczylas, Xiaoxiao Chen, Toni L. Cordero, John Shin, Ohad Cohen

**Affiliations:** 1 Department of Internal Diseases and Diabetology, Medical University of Lodz, Pomorska Str. 251, 92-213 Lodz, Poland; 2 Department of Reproduction, Poznan University of Medical Sciences, Poznań, Poland; 3 Department of Metabolic Diseases, The University Hospital in Krakow, Krakow, Poland; 4 Collegium Medicum, Jagiellonian University of Krakow, Krakow, Poland; 5 Medtronic, Warsaw, Poland; 6 Medtronic, Northridge, CA USA; 7 Medtronic, Tolochenaz, Switzerland

**Keywords:** Type 1 diabetes-complicated pregnancy, HbA1c, Continuous subcutaneous insulin infusion (CSII), Continuous glucose monitoring (CGM), Large for gestational age (LGA), Neonatal outcomes

## Abstract

**Aims:**

The effects of continuous subcutaneous insulin infusion (CSII) therapy with or without continuous glucose monitoring (CGM) on neonatal outcomes and glycemic outcomes of pregnant women with type 1 diabetes (T1D), living in Poland, were assessed.

**Methods:**

This prospective observational study enrolled women with *T*1D (*N* = 481, aged 18–45 years) who were pregnant or planned pregnancy. All used CSII therapy and a subset used CGM with CSII (CSII + CGM). Neonatal outcomes (e.g., rate of large for gestational age [LGA] delivery [birth weight > 90th percentile]) and maternal glycemia (e.g., HbA1c and percentage of time at sensor glucose ranges) were evaluated.

**Results:**

Overall HbA1c at trimesters 1, 2, and 3 was 6.8 ± 1.1% (50.9 ± 12.3 mmol/mol, *N* = 354), 5.8 ± 0.7% (40.1 ± 8.0 mmol/mol, *N* = 318), and 5.9 ± 0.7% (41.4 ± 8.0 mmol/mol, *N* = 255), respectively. A HbA1c target of < 6.0% (42 mmol/mol) at each trimester was achieved by 20.9% (74/354), 65.1% (207/318), and 58.0% (148/255), respectively. For women using CSII + CGM versus CSII only, HbA1c levels at trimesters 1, 2, and 3 were 6.5 ± 0.9% versus 7.1 ± 1.3% (47.8 ± 9.7 mmol/mol versus 54.3 ± 14.0 mmol/mol, *p* < 0.0001), 5.7 ± 0.6% versus 6.0 ± 0.9% (38.9 ± 6.5 mmol/mol versus 41.6 ± 9.3 mmol/mol, *p* = 0.0122), and 5.8 ± 0.6% versus 6.1 ± 0.8% (40.3 ± 6.9 mmol/mol versus 42.9 ± 9.1 mmol/mol, *p* = 0.0117), respectively. For the overall, CSII only, and CSII + CGM groups, rates of LGA delivery were 22.7% (74/326), 24.6% (34/138), and 21.3% (40/188), respectively.

**Conclusions:**

Observational assessment of women with *T*1D using CSII therapy demonstrated low HbA1c throughout pregnancy and low rates of LGA. The addition of CGM to CSII therapy compared to CSII therapy alone was associated with some improved maternal glycemic and neonatal outcomes.

**Clinicaltrials.gov identifier:**

NCT01779141 (January 2013).

**Supplementary Information:**

The online version contains supplementary material available at 10.1007/s00592-022-02020-9.

## Introduction

Diabetes management technology (i.e., continuous subcutaneous insulin infusion [CSII] therapy or continuous glucose monitoring [CGM]) has been shown to improve outcomes in non-pregnant individuals living with *T*1D [1–5]. Pregnancies complicated by *T*1D warrant particularly careful management, given the risks of adverse outcomes for both mother and baby [6]. Compared to pregnant women without diabetes, women with *T*1D are at a particularly increased risk of hypoglycemia (especially in early pregnancy) [7], diabetic ketoacidosis [8], preeclampsia [9], miscarriage [10, 11], preterm birth [12], and other complications [10, 13]. Compared to neonates of women without diabetes, babies of mothers with *T*1D are at increased risk of negative neonatal composite outcomes including being large for gestational age (LGA, birth weight > 90th percentile for the population) [11, 14], macrosomia (neonate birth weight > 4000 g, regardless of gestational age) [10, 15] or congenital malformation [12, 16], and having increased rates of perinatal mortality [11, 12, 16] and neonatal hypoglycemia [10, 11]. A majority of these negative neonatal outcomes are primarily associated with increased fetal hyperglycemia exposure [17–21]. In consequence, macrosomia and LGA affect about half of all babies of mothers with *T*1D [14], and notably these children go on to live with increased risk of future obesity [19], diabetes [17], and cardiovascular disease [22].

Fortunately, pre-pregnancy planning that includes either CSII or MDI therapy has been reported to improve HbA1c and pregnancy outcomes [23, 24] and, significantly, compared to unplanned pregnancy [23]. Recent prospective observational study of maternal and neonatal outcomes in pregnant women receiving comprehensive preconception planning with either CSII or MDI demonstrated significantly reduced severe adverse pregnancy outcomes when compared with women who received routine care [25]. While good or improved glycemic control (a low or lowered HbA1c level) is observed with intensive insulin treatment and/or diabetes technology management therapies, high rates of LGA or macrosomia [21, 26] and other complications can still persist.

Given the reported impact of pre-pregnancy planning and CGM therapy in pregnancies complicated by *T*1D [21, 27–29], we report on the maternal glycemic and neonatal composite outcomes from an observational study of pregnant women with T1D who used CSII with or without CGM during the Orchestra Foundation (Fundacja Wielka Orkiestra Swiatecznej Pomocy) insulin pump donation program in Poland.

## Methods

This prospective, observational study assessed Polish women (18–45 years of age) with pregestational *T*1D, who were pregnant or who planned pregnancy, and who were on MDI therapy for at least three months, before study start. Detailed inclusion and exclusion criteria of participants enrolled across 22 investigational centers throughout Poland have been published elsewhere [30]. Briefly, women with T1D were eligible to take part in the study if their physician recommended and prescribed CSII therapy or sensor-augmented insulin pump (SAP) therapy due to pregnancy or a plan to become pregnant. The decision to start CSII only or CSII with CGM (CSII + CGM) was made by the physician and study participant, independent of the study. Prior enrollment in and/or withdrawal from the Orchestra Foundation registry, current participation or participation in any other interventional clinical trial within the prior 3 months, in vitro fertilization assistance, pregnancy beyond 16 weeks of amenorrhea, any diabetes other than *T*1D, and inability to read or write were study exclusion criteria.

This study was conducted in compliance with the Declaration of Helsinki of 2008 by means of an informed consent process, Medical Ethics Committee approval (15/KBL/OIL/2013), study training, and clinical trial registration (CinicalTrials.gov identifier NCT01779141). The summary of study visits and points of data collection, including pump and blood glucose meter data uploads to CareLink™ clinical software (Medtronic, Northridge, California, USA), are listed in Supplementary Information S1. The informed consent form was signed at either the first (preconception) visit or first visit within the first trimester of pregnancy. The study protocol was approved by a Central Ethics Committee (Regional Chamber of Physicians at Krakow) for all investigational sites. Competent Authority approval was not required for observational studies in Poland. A STROBE cohort checklist [31] was completed for this report.

The Orchestra Foundation funded the CSII therapy for study participants. Approximately 60% were provided a MiniMed™ Paradigm™ REAL-Time 722 insulin pump (Medtronic) with or without a MiniMed™ Sof-sensor™ senor (Medtronic), and the remainder were provided a MiniMed™ Paradigm™ Veo™ system with the Enlite™ sensor (Medtronic). Participants provided the SAP system were not required to use the low-glucose suspend function.

Women participating in this study received intensive diabetes management that involved education and/or training on diet and carbohydrate counting, physical activity, folic acid supplementation, glycemic goals, self-monitoring of blood glucose (SMBG), and insulin dose adjustment, pump and CGM management (e.g., system calibrations and glucose sensor and infusion set changes). Care included frequent outpatient visits, and hospitalization if necessary. Participants underwent regular routine follow-up, without special requirements or regimens, which involved standard medical information collection into electronic clinical report forms and occasional data upload from devices (pumps and blood glucose meters) into CareLink™ clinical software (Medtronic). The study duration for each participant was up to 22 months (i.e., up to 12 months pre-conception and, if applicable, up to six weeks after delivery).

### Statistical and safety outcomes analyses

The primary objective was to assess the effect of CSII therapy with or without CGM on the HbA1c of overall women and those who enrolled before and during pregnancy, in general. Secondary objectives included assessment of maternal glycemic outcomes (i.e., sensor glucose [SG], standard deviation [SD] of SG, coefficient of variation [CV] of SG, and the percentage of time spent at SG ranges [32]) during CSII CSII + CGM use. Exploratory post hoc analysis of HbA1c was performed during CSII only therapy versus CSII + CGM. Analyses were conducted with two-sample t test or Wilcoxon rank-sum test (for categorical data), and a *p* < 0.05 was considered statistically significant. Missing data may have not been available due to a study participant forgetting to upload to CareLink™ clinical or not uploading successfully due to a technical issue. However, no imputation was applied for missing data in this study. Data were not adjusted for confounding.

The prevalence of pregnancy complications (i.e., large for gestational age [LGA], macrosomia, jaundice, congenital malformation, and neonatal death]) and admissions to the neonatal intensive care unit were recorded. LGA was based on Polish percentile grids that calculated neonatal weight in relation to a newborn’s sex [33]. Pregnancy dating was calculated according to first trimester ultrasound scan (i.e., crown-rump length between 30 and 84 mm, which corresponds with the 9 + 5–14 + 1 weeks of pregnancy [according to https://fetalmedicine.org/research/pregnancyDating]).

Exploratory post hoc analysis assessed the association of maternal SG, SD of SG, percentage of time spent at target SG range (63–140 mg/dL [3.5–7.8 mmol/L]) and 1-h postprandial SG [34, 35] with non-LGA versus LGA delivery and positive versus negative composite neonatal outcomes. An odds ratio of mean (95% confidence interval) was used to determine the strength of association and was analyzed with logistic regression. A *p* < 0.05 was considered statistically significant. Positive neonatal composite outcomes were defined as deliveries that were without congenital malformation, did not require mechanical ventilation, and were not LGA. Negative neonatal outcomes deliveries involved congenital malformation, mechanical ventilation requirement, LGA delivery or admittance to the neonatal care unit.

Due to the observational study design, methods to reduce bias were limited and analyses were conducted with respect to intention to treat. Safety outcomes were summarized as the incidence of maternal severe hypoglycemia (hypoglycemia requiring third-party assistance); diabetic ketoacidosis; unanticipated adverse device effects (UADEs, any serious adverse device effect which by its nature, incidence, severity or outcome had not been identified in the current version of the risk analysis report); serious adverse events (SAEs, defined as an adverse event that led to death, serious deterioration in the health of the subject that either resulted in a life-threatening illness or injury, or a permanent impairment of a body structure or a body function, or hospitalization); a medical or surgical intervention to prevent life-threatening illness or injury or permanent impairment to a body structure or a body function; or fetal distress, fetal death, congenital abnormality or birth defect. In the present study, severe hypoglycemia and diabetic ketoacidosis were treated as severe adverse events. The SAE and UADE information was collected throughout the study and reported immediately to Medtronic Poland (Sp.z.o.o) via an SAE/UADE form incorporated in the electronic clinical record.

## Results

A flow diagram (Supplementary Information S2) of participant disposition shows that a total of 481 women (mean age of 30.4 ± 4.0 years and diabetes duration of 12.3 ± 7.7 years) enrolled in the study; 216 of whom were not pregnant. There were 121 who dropped out or were withdrawn before pregnancy and 60 who dropped out or were withdrawn during pregnancy: the latter included 28 who miscarried, 10 early withdrawals prior to delivery, and 22 who gave birth but were lost to follow-up. Of the total 360 pregnant women, 190 (52.8%) used CGM with CSII therapy.

Table 1 summarizes the age, glycemic metrics, total daily insulin, and medical history of the overall group, at baseline. The same characteristics are shown for those who used CSII only or CSII + CGM. The rate of complications (macroangiopathy, nephropathy, neuropathy, and retinopathy) is also provided for each of the aforementioned groups.

**Table 1:** Summary of age, glycemic metrics, total daily insulin, and medical history at baseline of study participants

	Overall (*N* = 360)	CSII only (*N* = 167)	CSII + CGM (*N* = 193)^d^
Age, years	30.1 ± 4.0	29.9 ± 4.3	30.3 ± 3.8
HbA1c^a^	(*N* = 354)	(*N* = 166)	(*N* = 188)
%	6.8 ± 1.1	7.1 ± 1.3	6.5 ± 0.9
mmol/mol	50.9 ± 12.3	54.3 ± 14.0	47.8 ± 9.7
SMBG^b^	(*N* = 202)	(*N* = 60)	(*N* = 142)
mg/dL	115.5 ± 16.6	115.7 ± 17.3	115.5 ± 16.3
mmol/L	6.4 ± 0.9	6.4 ± 1.0	6.4 ± 0.9
TDD, units	43.5 ± 15.4	44.5 ± 14.1	42.6 ± 16.4
Weight, kg	65.3 ± 11.5	66.0 ± 12.6	64.7 ± 10.5
BMI, kg/m^2^	23.7 ± 3.7	23.9 ± 3.8	23.5 ± 3.6
Diabetes history, years	12.0 ± 7.8	12.1 ± 7.8	11.9 ± 7.9
Previous severe hypoglycemia, *N*^c^	0.1 ± 0.5	0.1 ± 0.5	0.1 ± 0.5
Previous DKA, *N*^c^	0.0 ± 0.2	0.0 ± 0.2	0.0 ± 0.2
Macroangiopathy, %	1.7 (6/360)	2.4 (4/167)	1.0 (2/193)
Nephropathy, %	3.9 (14/360)	6.0 (10/167)	2.1 (4/193)
Neuropathy, %	5.6 (20/360)	6.6 (11/167)	4.7 (9/193)
Retinopathy, %	15.3 (55/360)	17.4 (29/167)	13.5 (26/193)

Values are shown as mean ± SD or mean. Numbers within parentheses indicate number of participants or participants over total number of participants.

^a^The first trimester HbA1c of women who enrolled during pregnancy (*N* = 262) was a mixture of HbA1c levels collected before enrollment (*N* = 206) and on or after enrollment (*N* = 56).

^b^The first trimester SMBG of women who enrolled during pregnancy was only collected after study enrollment and may not represent the averaged metric for the whole trimester.

^**c**^Up to 12 months prior to study start.

^d^Three women did not become pregnant but provided baseline enrollment data.

*HbA1c* glycated hemoglobin, *SMBG *self-monitoring of blood glucose, *TDD* total daily dose of insulin, *DKA* diabetic ketoacidosis

### Maternal glucose outcomes

Mean HbA1c at each trimester is shown for the overall group and those who enrolled before or during pregnancy, stratified by therapy use (Table 2). Women who enrolled during pregnancy made up a significant proportion of the overall group and their mean HbA1c at *T*1 trended higher (*N* = 262, 7.0 ± 1.2% [53.1 ± 12.9 mmol/mol]) than that of women who enrolled before pregnancy (*N* = 92, 6.2 ± 0.7% [44.5 ± 7.7 mmol/mol]). By *T*3, however, mean HbA1c for both groups (5.9 ± 0.7% [41.2 ± 8.1 mmol/mol] and 6.0 ± 0.7% [41.9 ± 7.7 mmol/mol], respectively) appeared relatively comparable. The use of CGM with CSII therapy by women who enrolled during pregnancy, compared with CSII alone, resulted in a statistically significant reduction in HbA1c over the course of pregnancy (Table 2). This trend was not observed in women who enrolled before pregnancy, although HbA1c reduced with time.

**Table 2:** HbA1c of groups who used CSII therapy only or CGM with CSII therapy

	Overall (*N*)			Enrolled before pregnancy (*N*)			Enrolled during pregnancy (*N*)		
	*T*1*	*T*2	*T*3	T1	*T*2	*T*3	*T*1*	*T*2	*T*3
**Total**									
HbA1c	(*N* = 354)	(*N* = 318)	(*N* = 255)	(*N* = 92)	(*N* = 79)	(*N* = 61)	(*N* = 262)	(*N* = 239)	(*N* = 194)
%	6.8 ± 1.1	5.8 ± 0.7	5.9 ± 0.7	6.2 ± 0.7	5.8 ± 0.6	6.0 ± 0.7	7.0 ± 1.2	5.8 ± 0.8	5.9 ± 0.7
mmol/mol	50.9 ± 12.3	40.1 ± 8.0	41.4 ± 8.0	44.5 ± 7.7	39.6 ± 6.6	41.9 ± 7.7	53.1 ± 12.9	40.2 ± 8.4	41.2 ± 8.1
**CSII only**									
HbA1c	(*N* = 166)	(*N* = 140)	(*N* = 108)	(*N* = 34)	(*N* = 27)	(*N* = 22)	(*N* = 132)	(*N* = 113)	(*N* = 86)
%	7.1 ± 1.3	6.0 ± 0.9	6.1 ± 0.8	6.4 ± 0.8	5.9 ± 0.6	6.1 ± 0.8	7.3 ± 1.3	6.0 ± 0.9	6.1 ± 0.9
mmol/mol	54.3 ± 14.0	41.6 ± 9.3	42.9 ± 9.1	46.2 ± 8.4	41.6 ± 6.8	43.0 ± 8.3	56.4 ± 14.4	41.7 ± 9.8	42.8 ± 9.3
**CSII + CGM**									
HbA1c	(*N* = 188)	(*N* = 178)	(*N* = 147)	(*N* = 58)	(*N* = 52)	(*N* = 39)	(*N* = 130)	(*N* = 126)	(*N* = 108)
%	6.5 ± 0.9	5.7 ± 0.6	5.8 ± 0.6	6.1 ± 0.7	5.7 ± 0.6	5.9 ± 0.7	6.7 ± 0.9	5.7 ± 0.6	5.8 ± 0.6
mmol/mol	47.8 ± 9.7	38.9 ± 6.5	40.3 ± 6.9	43.5 ± 7.1	38.6 ± 6.3	41.3 ± 7.4	49.7 ± 10.1	39.0 ± 6.6	39.9 ± 6.7
*p*-value	< 0.0001^b^	0.0122^b^	0.0117^b^	0.0471^b^	0.0619^a^	0.6355^b^	< 0.0001^b^	0.0399^b^	0.0097^b^

Values are shown as mean ± SD.

*P*-values indicate difference between CSII only and CSII + CGM within each given trimester.

*The first trimester HbA1c of women who enrolled during pregnancy (*N* = 262) was a mixture of HbA1c levels collected before enrollment (*N* = 206) and on or after enrollment (*N* = 56).

*T*1, *T*2, *T*3 = Trimester 1, 2, and 3, respectively; HbA1C = glycated hemoglobin; *CSII *continuous subcutaneous insulin infusion; *CGM* continuous glucose monitoring.

^a^Two-sample t-test.

^b^Wilcoxon rank-sum test.

The proportion of women from all groups achieving mean HbA1c ranges throughout pregnancy, including the mean target HbA1c of < 6.0% (< 42.0 mmol/mol) [34, 35], is stratified by therapy use and shown in Supplementary Information S3. The percentage achieving target HbA1c tended to increase from trimesters 1 to 3 and were 20.9% (74/354), 65.1% (207/318), and 58.0% (148/255), respectively. By trimester 3, the proportion who achieved target HbA1c with CSII only was 49.1% (*N* = 53/108), while that for women who used CGM with CSII was 64.6 (*N* = 95/147).

Table 3 shows the CGM outcomes of all women who used CGM with CSII therapy. Average SG for all groups was < 120 mg/dL (< 6.7 mmol/L), CV of SG averaged < 33%, and a trending increase in time spent in target range from the first to the third trimester was observed. While time in hypoglycemic ranges (< 63 mg/dL [< 3.5 mmol/mol] and < 54 mg/dL [< 3.0 mmol/mol]) still averaged more than the targeted < 1 h/day and < 0.25 h/day, respectively, time in target range reached > 70% and the proportion of time spent at > 140 mg/dL (> 7.8 mmol/mol) remained below 25%, for all groups.

**Table 3:** Summary of glycemic outcomes throughout pregnancy in women who used CGM with CSII therapy

	Overall (*N*)			Enrolled before pregnancy (*N*)			Enrolled during pregnancy (*N*)		
	*T*1^*^ (*N* = 109)	*T*2 (*N* = 167)	*T*3 (*N* = 151)	*T*1 (*N* = 50)	*T*2 (*N* = 49)	*T*3 (*N* = 47)	*T*1^*^ (*N* = 59)	*T*2 (*N* = 118)	*T*3 (*N* = 104)
SG, mg/dL	113.0 ± 16.5	111.7 ± 14.7	113.7 ± 13.7	116.6 ± 17.5	113.1 ± 14.3	115.9 ± 11.6	109.9 ± 15.1	111.1 ± 14.9	112.7 ± 14.5
SG, mmol/L	6.3 ± 0.9	6.2 ± 0.8	6.3 ± 0.8	6.5 ± 1.0	6.3 ± 0.8	6.4 ± 0.6	6.1 ± 0.8	6.2 ± 0.8	6.3 ± 0.8
CV of SG, %	35.1 ± 7.6	33.6 ± 7.4	31.2 ± 5.6	34.5 ± 6.7	32.5 ± 5.5	30.1 ± 5.3	35.7 ± 8.2	34.1 ± 8.0	31.7 ± 5.6
*Percentage of time spent at sensor glucose ranges, mg/dL*									
< 54	3.1 ± 4.1	2.9 ± 3.4	2.1 ± 2.8	2.3 ± 2.4	2.3 ± 2.5	1.4 ± 1.6	3.7 ± 5.0	3.2 ± 3.6	2.3 ± 3.2
< 63	7.6 ± 6.5	7.2 ± 5.6	5.2 ± 5.0	6.3 ± 5.2	6.0 ± 4.6	3.8 ± 3.0	8.7 ± 7.3	7.7 ± 6.0	5.9 ± 5.5
63–140	70.4 ± 12.4	72.2 ± 11.7	73.9 ± 11.0	69.2 ± 13.2	72.4 ± 10.7	74.3 ± 10.5	71.3 ± 11.7	72.2 ± 12.1	73.7 ± 11.2
> 140	22.1 ± 13.0	20.6 ± 12.0	20.9 ± 11.6	24.5 ± 14.1	21.5 ± 11.9	21.9 ± 10.8	20.1 ± 11.8	20.1 ± 12.0	20.4 ± 12.0
> 250	1.2 ± 2.0	0.8 ± 2.4	0.6 ± 1.1	1.4 ± 2.5	0.6 ± 1.1	0.5 ± 0.7	1.0 ± 1.4	0.9 ± 2.7	0.6 ± 1.2

Values are shown as mean ± SD.

*The first trimester SG of women who enrolled during pregnancy was only collected after study enrollment and may not represent the averaged metric for the whole trimester.

*T*1, *T*2, *T*3 = Trimester 1, 2, and 3, respectively; *SG* sensor glucose; *CV* coefficient of variation.

### Neonatal status and outcomes

For the overall group and those who enrolled before or during pregnancy, a summary of neonatal status (i.e., sex, gestational delivery, mean birth length and weight), rates of blood glucose levels ≤ 40 mg/dL, and prevalence of complications ([e.g., large for gestational age, macrosomia, jaundice, congenital malformation, and death]) are detailed in Supplementary Information S4. Briefly, the rates of LGA (birth weight > 90th percentile) were 22.7% (74/326), 28.0% (23/82), and 20.9% (51/244) for each respective group. For women using only CSII, the rate was 24.6% (34/138), and for those using CGM with CSII it was 21.3% (40/188). Rates of neonatal hypoglycemia (BG ≤ 40 mg/dL) were also low across groups and were 20.1% (51/254), 17.9% (10/56), and 20.7% (41/198), respectively, and appeared relatively comparable between women who used CSII only (21.0% [21/100]) and CGM with CSII (19.5% [30/154]). This trend was also observed for Caesarean births, where women who used CGM demonstrated a rate of 22% (42/189) and those who used only CSII demonstrated a rate of 24.5% (34/139). The exploratory analysis of non-LGA versus LGA delivery and positive versus negative neonatal composite outcomes with respect to maternal CGM metrics demonstrated strong association with most of the recommended glycemic targets for pregnancy (Table 4).

**Table 4:** Association of maternal glycemic and neonatal outcomes during CGM use with CSII therapy

Non-LGA delivery	SG, mg/dL	SD of SG, mg/dL	Time in target range, %	1-hour postprandial SG, mg/dL		
				*T*1	*T*2	*T*3
Non-LGA delivery	111.1 ± 14.5 (*N*=140)	37.6 ± 11.4 (*N = *140)	73.0 ± 11.3 (*N = *140)	113.8 ± 21.3 (*N = *46)	112.1 ± 19.6 (*N = *93)	110.5 ± 17.5 (*N = *81)
LGA delivery	119.9 ± 11.7 (*N = *40)	40.3 ± 8.5 (*N = *40)	67.9 ± 9.4 (*N = *40)	125.9 ± 17.5 (*N = *14)	123.6 ± 16.8 (*N = *26)	120.3 ± 13.0 (*N = *24)
Odds ratio	1.04 [1.02, 1.07]	1.02 [0.99, 1.05]	0.96 [0.93, 0.99]	1.03 [1.00, 1.06]	1.03 [1.01, 1.05]	1.04 [1.01, 1.07]
*p*-value	0.0002^b^	0.0456^b^	0.0045^b^	0.0578^a^	0.0014^b^	0.0119^a^
Positive composite neonatal outcomes^*^	110.3 ± 14.2 (*N *= 126)	36.7 ± 8.7 (*N = *126)	73.7 ± 10.8 (*N = *126)	113.6 ± 21.5 (*N = *42)	112.3 ± 19.6 (*N = *86)	109.8 ± 17.6 (*N = *77)
Negative composite neonatal outcomes^†^	119.5 ± 12.6 (*N = *54)	41.9 ± 14.0 (*N = *54)	67.5 ± 10.7 (*N = *54)	123.5 ± 18.3 (*N = *18)	120.8 ± 18.3 (*N = *33)	120.6 ± 12.4 (*N = *28)
Odds ratio	1.05 [1.02, 1.08]	1.05 [1.01, 1.09]	0.95 [0.92, 0.98]	1.02 [1.00, 1.05]	1.02 [1.00, 1.04]	1.04 [1.01, 1.07]
*p*-value	<0.0001^b^	0.0096^b^	0.0005^a^	0.0946^a^	0.0078^b^	0.0008^a^
Positive total composite neonatal outcomes^**^	109.9 ± 13.3 (*N = *120)	36.6 ± 8.5 (*N = *120)	73.8 ± 10.4 (*N = *120)	113.5 ± 20.9 (*N = *40)	111.9 ± 19.3 (*N = *83)	109.6 ± 16.7 (*N = *73)
Negative total composite neonatal outcomes^††^	119.5 ± 14.3 (*N = *60)	41.6 ± 13.9 (*N = *60)	67.9 ± 11.4 (*N = *60)	122.8 ± 20.1 (*N = *20)	121.0 ± 19.0 (*N = *36)	119.7 ± 15.7 (*N = *32)
Odds ratio	1.05 [1.03, 1.08]	1.05 [1.01, 1.09]	0.95 [0.92, 0.98]	1.02 [1.00, 1.05]	1.02 [1.00, 1.05]	1.04 [1.01, 1.07]
*p*-value	< 0.0001^a^	0.0140^b^	0.0006^a^	0.0557^b^	0.0048^b^	0.0046^a^

Values are shown as mean ± SD.

Time in target range = 63–140 mg/dL (3.5–7.8 mmol/L).

Odds ratios are shown with 95% confidence intervals.

^*^Deliveries that involved no congenital malformation, did not require mechanical ventilation, and were not LGA.

^**^Deliveries that involved no congenital malformation, did not require mechanical ventilation, were not LGA, and required no admittance to neonatal care unit.

^†^Deliveries that involved congenital malformation, required mechanical ventilation, or were LGA.

^††^Deliveries that involved congenital malformation, required mechanical ventilation, were LGA, or required admittance to neonatal care unit.

*T*1,*T* 2, *T*3 = Trimester 1, 2, and 3, respectively; *LGA * large for gestational age; *SG* sensor glucose.

^a^Two-sample t-test.

^b^Wilcoxon rank-sum test.

### Safety analyses

Regarding overall safety outcomes, there were 377 adverse events and 364 SAEs (Supplementary Information S5). Of the 364 SAEs, 97 were in the group that enrolled before pregnancy and 267 were in the group that enrolled during pregnancy. There were 357 non-device-related events that included three episodes of DKA and seven device-related events that included three episodes of DKA. There were eight episodes of severe hypoglycemia; three were in the group that enrolled before pregnancy and five were in the group that enrolled during pregnancy. All episodes of severe hypoglycemia occurred in participants who used CGM with CSII therapy. There were no reports of UADEs. There was one report of maternal death due to chronic kidney disease, 6 months after delivery.

## Discussion

This observational study shows that CSII therapy helped women with *T*1D who were pregnant or planning pregnancy achieve glycemic targets throughout pregnancy. Specifically, HbA1c levels of the overall group were reduced from 6.8 ± 1.1% (50.9 ± 12.3 mmol/mol) at trimester 1 to 5.8 ± 0.7% (40.1 ± 8.0 mmol/mol) and 5.9 ± 0.7% (41.4 ± 8.0 mmol/mol) at trimesters 2 and 3, respectively. There was also a trending increase in the proportion of women achieving target HbA1c by the third trimester. For the group who enrolled during pregnancy and used CGM with CSII, mean HbA1c was significantly reduced over trimesters, when compared to the HbA1c of women using only CSII. The significant reduction in HbA1c, with time, may not have been observed in women who enrolled before pregnancy due to the smaller sample size, as well as a smaller HbA1c difference between the CSII only and CGM + CSII therapies, at study start. For instance, this group demonstrated near-target HbA1c at T1, compared with the HbA1c of women who enrolled during pregnancy. While they also spent a lower percentage of time in hypoglycemia, all women using CGM with CSII spent time in target range that exceeded > 70% by the third trimester.

Exploratory analyses showed that achieving lower HbA1c and increased time in target glycemic range was associated with better neonatal outcomes. For the overall group of women, those who used CSII only and those who used CGM with CSII, the LGA delivery incidence rates were lower than other previously reported rates that reached ≥ 50%, in pregnant women with pregestational *T*D [21, 36]. When evaluated against the World Health Organization criteria [37], the LGA rates for each aforementioned group were 35.3%, 39.9%, and 31.9%, respectively.

Several organizations have recommended targeted diabetes management for pregnancies complicated by *T*1D: an HbA1c target of < 6.0% (< 42 mmol/mol) [34, 35], a fasting glucose level of < 90 mg/dL (< 5.0 mmol/L) [35] or < 95 mg/dL (< 5.3 mmol/L) [34], and a 1-h postprandial glucose concentration of < 140 mg/dL (< 7.8 mmol/L) [34, 35]. These metrics worsen in the later trimesters of pregnancy where challenges with gastric emptying and insulin kinetics [38] and impaired glucose metabolism [39] result in increased time above SG target range [20, 21, 28]. In the present study, however, these metrics met or aligned closely with consensus recommendations (e.g., average SG, 1-h postprandial SG of < 120 mg/dL, and CV of SG < 36%) [34, 40].

Prospectively randomized [28, 29, 41] and retrospective [21, 42] studies of CSII and/or CGM use versus MDI and SMBG measurements have demonstrated clinical benefits for mothers and their babies. Although the proportion of time spent in target glucose range increased and HbA1c improved (or did not change) with CGM technology relative to control [21, 28], only a small percentage of study participants reached the international consensus recommended TIR of > 70% (~ 17 h of the day). In addition, the overall rates of LGA deliveries in some of these trials remained rather high, averaging close to 50% in the intervention groups and up to 70% in the control groups. A recent observational study of pregnant women with T1D (*N* = 81, 11 on MDI and 70 on CSII [28 used CGM]), some of whom underwent pregnancy planning, demonstrated significantly improved HbA1c in those who used CGM with CSII compared to CSII or MDI therapy only (time spent at sensor glucose ranges was not analyzed) [27]. However, there was no difference in macrosomia risk between groups and the rate observed for women using CGM + CSII was 21.4%, but 16.7% for women using CSII only. In the present study, rates of macrosomia (weight of ≥ 4000 g) trended similarly and were 22.8% (43/189) for women using CGM + CSII and 18.0% (25/139) for those using only CSII.

Limitations of the current study include its observational and non-randomized design, and the relatively good glycemia management in study participants before and/or during the first trimester, which may preclude generalizability of findings. In addition, a majority of participants received multidisciplinary clinical support from gynecologists/obstetricians, diabetologists, nurses, and dieticians during the study. Similar findings of significantly reduced adverse pregnancy outcomes during prospective observational [25] and retrospective [43] studies have been reported in women with ideally managed T1D receiving comprehensive preconception-to-pregnancy planning or healthcare management. Thus, a clear distinction between the contribution of technological devices and a comprehensive multidisciplinary approach to diabetes management during pregnancy cannot be easily delineated. Indeed, the observational design was intended to bench mark the achievements of care in diabetes pregnancy clinics that used diabetes technology, not provide specific comparative outcomes. Strengths of this study include its report of outcomes observed in over 300 pregnancies complicated by T1D in women who demonstrated good compliance, enrolled pre- and postconception, and for whom HbA1c and CGM data could be analyzed.

## Conclusions

This observational investigation determined that good maternal glycemia and positive neonatal outcomes can be achieved in a majority of women who were pregnant or planned pregnancy and used CSII only or CGM with CSII. Maternal HbA1c and glucose levels improved throughout the course of pregnancy and positive neonatal composite outcomes were associated with an increased duration of maternal time spent within target glucose range. Although current insulin delivery therapies have advanced to include predictive insulin suspension and/or automated insulin delivery based on CGM, present study findings serve as a benchmark for pregnancy outcomes in women with T1D using only CSII or CGM with CSII.

## Supplementary Information

Below is the link to the electronic supplementary material.

Supplementary file1 (DOCX 429 kb)

## Data Availability

Study data can be accessed via this manuscript and its supplementary information or materials.
