# Supplementary material for: Insulin pump therapy with and without continuous glucose monitoring in pregnant women with type 1 diabetes: a prospective observational Orchestra Foundation study in Poland
Source: Acta Diabetol. 2023 Jan 19;60(4):553–61. doi: 10.1007/s00592-022-02020-9 (PMC10033617; doi:10.1007/s00592-022-02020-9)
Supplement: Supplementary file 1 — Supplementary file1 (DOCX 429 kb) [file 592_2022_2020_MOESM1_ESM.docx]

# Supplementary Information

**Journal**: Acta Diabetologica

**Title:** Insulin pump therapy with and without continuous glucose monitoring in pregnant women with type 1 diabetes: A prospective observational Orchestra Foundation study in Poland

**Authors**: Katarzyna Cypryk^1^, Prof; Ewa Wender-Ozegowska^2^, Prof; Katarzyna Cyganek^3,4^, Prof; Jacek Sieradzki^4^, Prof; Kinga Skoczylas^5^, MD; Xiaoxiao Chen^6^, PhD; Toni L. Cordero^6^, PhD; John Shin^6^, Ohad Cohen^8^, MD

**Author Affiliations**:

^1^Department of Internal Medicine and Diabetology, Medical University of Lodz, Poland
^2^Department of Reproduction, Poznan University of Medical Sciences, Poland
^3^Department of Metabolic Diseases, The University Hospital in Krakow, Poland
^4^Collegium Medicum, Jagiellonian University of Krakow, Poland
^5^Medtronic, Warsaw, Poland
^6^Medtronic, Northridge, California, USA

^7^Medtronic, Tolochenaz, Switzerland

**Corresponding Author**:

Katarzyna Cypryk

Department of Internal Diseases and Diabetology

Medical University of Lodz

Pomorska Str. 251, 92-213 Lodz, Poland

**Office:** +(48) 42 201 43 40

**E-mail:** katarzyna.cypryk@umed.lodz.pl

Supplementary Information (S1) Summary of study visits and data collection

| **Visit Number** | Visit 1  Preconception | Visit 2a^1^ | Visit 2b^2^ | Visit 1  Pregnancy | Visit 3 | Visit 4 | Visit 5^3^ | Visit 6 | Early  Study End |
| --- | --- | --- | --- | --- | --- | --- | --- | --- | --- |
| **Target Period** | Preconception  Planning | 12 weeks Amenorrhea  (±4 weeks) | 12 months  (±2 weeks) | Pregnancy | 24 weeks Amenorrhea  (±2 weeks) | 36 weeks Amenorrhea  (±2 weeks) | Delivery  (+2 weeks) | 6 weeks After Delivery  (±2 weeks) | NA |
| **Procedure** |  |  |  |  |  |  |  |  |  |
| **Patient Informed Consent** | X |  |  | X |  |  |  |  |  |
| **Inclusion/exclusion Criteria** | X |  |  | X |  |  |  |  |  |
| **ID Number Assignment** | X |  |  | X |  |  |  |  |  |
| **Complete HFS Questionnaire** | X |  | X | X | X | X |  | X |  |
| **Complete DTSQs** | X |  | X | X | X | X |  | X |  |
| **Complete DTSQc** |  |  |  |  | X |  |  |  |  |
| **Complete Demographics eCRF** | X |  |  | X |  |  |  |  |  |
| **Create CareLink™ Software**  **Account** | X |  |  | X |  |  |  |  |  |
| **Upload Pump Data to CareLink™**  **Software** |  | X | X |  | X | X | X | X |  |
| **Upload BG Meter to CareLink™**  **Software** |  | X | X |  | X | X | X | X |  |
| **Verify Patient Proper Device**  **Training** | X |  |  | X |  |  |  |  |  |
| **Record Distributed Devices** | X |  |  | X |  |  |  |  |  |
| **Distribute Supplies** | X | X |  | X | X | X | X | X |  |
| **Complete Follow-up eCRF** |  | X | X |  | X | X | X | X |  |
| **Collect SAEs** |  | X | X |  | X | X | X | X | X |
| **Complete End of Study eCRF** |  |  | If No Pregnancy |  |  |  |  |  | X |
| **Return of Devices** |  |  | If No Pregnancy |  |  |  |  | X | X  Early Dropout |

^1^If patient was pregnant at enrollment, “Visit 1-Pregnancy” procedures were followed.

^2^For women who became pregnant within six months of follow up, ”Visit 2b” was not applicable.

^3^Data collected in eCRF at two weeks or during “Visit 6”.

Early dropout/withdrawal or miscarriage involved the following procedures: collection of serious adverse events, completion of end of study eCRF and return of device.

HFS=Hypoglycemia Fear Survery, DTSQs=Diabetes Treatment Satisfaction Qustionnaire (status), Diabetes Treatment Satisfaction Questionnaire (change), eCRF=electronic Clinical Report Form, BG=Blood Glucose, SAEs=Serious Advere Events.

Supplementary Information (S2) Disposition and flow diagram


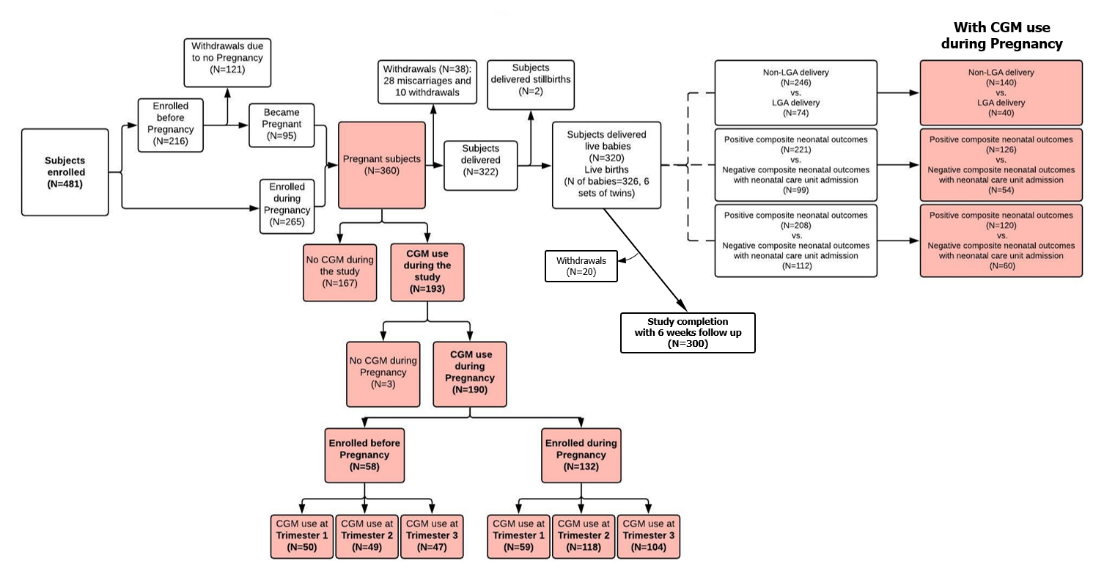


**S2 Caption** The flow diagram shows participant disposition from study enrollment through study completion

Supplementary Information (S3) Proportion of women achieving mean HbA1c levels throughout pregnancy, stratified by therapy

| **HbA1c, %**  **(mmol/mol)** | **Proportion achieving HbA1c level, %**  **(N/Total N)** | | |
| --- | --- | --- | --- |
|  | **T1^1^** | **T2** | **T3** |
| **Total** | | | |
| **<6.0**  **(<42.0)** | 20.9  (74/354) | 65.1  (207/318) | 58.0  (148/255) |
| **<6.5**  **(<48.0)** | 47.5  (168/354) | 84.9  (270/318) | 79.2  (202/255) |
| **<7.0**  **(<53.0)** | 64.4  (228/354) | 94.3  (300/318) | 93.3  (238/255) |
| **<7.5**  **(<58.0)** | 77.4  (274/354) | 97.5  (310/318) | 98.0  (250/255) |
| **<8.0**  **(<64.0)** | 85.9  (304/354) | 98.7  (314/318) | 99.2  (253/255) |
| **CSII only** | | | |
| **<6.0**  **(<42.0)** | 13.3  (22/166) | 57.1  (80/140) | 49.1  (53/108) |
| **<6.5**  **(<48.0)** | 36.1  (60/166) | 80.0  (112/140) | 75.0  (81/108) |
| **<7.0**  **(<53.0)** | 53.6  (89/166) | 90.0  (126/140) | 90.7  (98/108) |
| **<7.5**  **(<58.0)** | 69.9  (116/166) | 95.0  (133/140) | 97.2  (105/108) |
| **<8.0**  **(<64.0)** | 79.5  (132/166) | 97.1  (136/140) | 98.1  (106/108) |
| **CSII + CGM** | | | |
| **<6.0**  **(<42.0)** | 27.7  (52/188) | 71.3  (127/178) | 64.6  (95/147) |
| **<6.5**  **(<48.0)** | 57.4  (108/188) | 88.8  (158/178) | 82.3  (121/147) |
| **<7.0**  **(<53.0)** | 73.9  (139/188) | 97.8  (174/178) | 95.2  (140/147) |
| **<7.5**  **(<58.0)** | 84.0  (158/188) | 99.4  (177/178) | 98.6  (145/147) |
| **<8.0**  **(<64.0)** | 91.5  (172/188) | 100.0  (178/178) | 100.0  (147/147) |

Values are shown as percentage (N/Total N)

^1^The first trimester HbA1c of women who enrolled during pregnancy (N=262) was a mixture of HbA1c levels collected before enrollment (N=206) and on or after enrollment (N=56).

T1, T2, T3=Trimester 1, 2, and 3, respectively; HbA1c=Glycated hemoglobin.

Supplementary Information (S4) Summary of neonatal delivery status and prevalence of neonatal complications

| **Category** | **Overall**  **(N=328)** | **Neonates of participants enrolled before pregnancy**  **(N=83)** | **Neonates of participants enrolled during pregnancy**  **(N=245)** |
| --- | --- | --- | --- |
| **Sex (male/female)** | 175/153 | 44/39 | 131/114 |
| **Gestational delivery, weeks** | 37.2±2.2 | 37.4±2.2 | 37.2±2.2 |
| **Length, cm** | 54.2±5.7 | 54.1±7.4 | 54.2±5.0 |
| **Weight, kg** | 3.4±0.8 | 3.6±0.8 | 3.3±0.7 |
| **Mean blood glucose, mg/dL** | 58.9±41.4 | 61.6±26.2 | 58.2±44.9 |
| **Prevalence of neonatal complications** | | | |
| **LGA** | 22.7%  (74/326) | 28.0%  (23/82) | 20.9%  (51/244) |
|  |  | CSII only: 28.6% (8/28)  CSII + CGM: 27.8% (15/54) | CSII only: 23.6% (26/110)  CSII + CGM: 18.7% (25/134) |
| **Weight <4,000 g** | 79.3%  (260/328) | 69.9%  (58/83) | 82.4%  (202/245) |
|  |  | CSII only: 78.6% (22/28)  CSII + CGM: 65.5% (36/55) | CSII only: 82.9% (92/111)  CSII + CGM: 82.1% (110/134) |
| **Weight 4,000-4,500 g** | 17.4%  (57/328) | 26.5%  (22/83) | 14.3%  (35/245) |
|  |  | CSII only: 17.9% (5/28)  CSII + CGM: 30.9% (17/55) | CSII only: 14.4% (16/111)  CSII + CGM: 14.2% (19/134) |
| **Weight >4,500 g** | 3.4%  (11/328) | 3.6%  (3/83) | 3.3%  (8/245) |
|  |  | CSII only: 3.6% (1/28)  CSII + CGM: 3.6% (2/55) | CSII only: 0.7% (3/111)  CSII + CGM: 3.7% (5/134) |
| **Blood glucose ≤40 mg/dL** | 20.1%  (51/254) | 17.9%  (10/56) | 20.7%  (41/198) |
|  |  | CSII only: 12.5% (2/16)  CSII + CGM: 20.0% (8/40) | CSII only: 22.6% (19/84)  CSII + CGM: 19.3% (22/114) |
| **Caesarean birth** | 23.2%  (76/328) | 24.1%  (20/83) | 22.9%  (56/245) |
|  |  | CSII only: 25.0% (7/28)  CSII + CGM: 23.6% (13/55) | CSII only: 24.3% (27/111)  CSII + CGM: 21.6% (29/134) |
| **Jaundice** | 57.7%  (188/326) | 54.9%  (45/82) | 58.6%  (143/244) |
|  |  | CSII only: 57.1% (16/28)  CSII + CGM: 53.7% (29/54) | CSII only: 53.6% (59/110)  CSII + CGM: 62.7% (84/134) |
| **Congenital malformation** | 6.7%  (22/328) | 3.6%  (3/83) | 7.8%  (19/245) |
|  |  | CSII only: 3.6% (1/28)  CSII + CGM: 3.6% (2/55) | CSII only: 8.1% (9/111)  CSII + CGM: 7.5% (10/134) |
| **Neonatal death** | 0.6%  (2/328) | 1.2%  (1/83) | 0.4%  (1/245) |
|  |  | CSII only: 0.0% (0/28)  CSII + CGM: 1.8% (1/55) | CSII only: 0.9% (1/111)  CSII + CGM: 0.0% (0/134) |
| **NCU admission** | 10.0%  (31/309) | 7.6%  (6/79) | 10.9%  (25/230) |
|  |  | CSII only:19.2% (5/26)  CSII + CGM: 1.9% (1/53) | CSII only: 14.0% (14/100)  CSII + CGM: 8.5% (11/130) |
| **Duration of NCU admission, days** | 11.7±9.5 | 8.8±10.6 | 12.4±9.3 |
|  |  | CSII only: 9.8±11.5  CSII + CGM: 4.0±0.0 | CSII only: 12.6±9.4  CSII + CGM: 12.1±9.7 |

Values are shown as mean or mean±SD, excluding sex. Data are based on full-term deliveries without miscarriage.

NCU=Neonatal care unit.

LGA=Large for gestational age (birth weight >90^th^ percentile).

CSII=Continuous subcutaneous insulin infusion.

CGM=Continuous glucose monitoring.

Supplementary Information (S5) Summary of adverse and serious adverse events for all groups

| **Category** | **Before  pregnancy** | **T1** | **T2** | **T3** | **After Delivery** | **Neonatal Adverse Events** | **Total** |
| --- | --- | --- | --- | --- | --- | --- | --- |
| **Overall** | | | | | | | |
| **Number of adverse events** | 14 | 40 | 45 | 150 | 4 | 124 | 377 |
| **Death** | 0 | 0 | 0 | 0 | 1 | 0 | 1 |
| **Device-related adverse events** | 4 | 0 | 3 | 1 | 0 | 0 | 8 |
| **Serious adverse events** | 13 | 39 | 44 | 147 | 4 | 117 | 364 |
| **^1^Severe hypoglycemic episodes** | 0 | 6 | 1 | 1 | 0 | 0 | 8 |
| **^2^Diabetic ketoacidosis** | 4 | 0 | 1 | 1 | 0 | 0 | 6, |
| **Enrolled before pregnancy** | | | | | | | |
| **Number of adverse events** | 14 | 16 | 7 | 36 | 2 | 26 | 101 |
|  | CSII only: 3  CSII + CGM: 11 | CSII only: 6  CSII + CGM: 10 | CSII only: 5  CSII + CGM: 2 | CSII only: 16  CSII + CGM: 20 | CSII only: 1  CSII + CGM: 1 | CSII only: 13  C SII + CGM: 13 | CSII only: 44  CSII + CGM: 57 |
| **Death** | 0 | 0 | 0 | 0 | 0 | 0 | 0 |
|  | -- | -- | -- | -- | -- | -- | -- |
| **Device-related adverse events** | 4 | 0 | 0 | 0 | 0 | 0 | 4 |
|  | CSII only: 1  CSII + CGM: 3 | -- | -- | -- | -- | -- | CSII only: 1  CSII + CGM: 3 |
| **Serious adverse events** | 13 | 16 | 6 | 36 | 2 | 24 | 97 |
|  | CSII only: 3  CSII + CGM: 10 | CSII only: 6  CSII + CGM: 10 | CSII only: 4  CSII + CGM: 2 | CSII only: 16  CSII + CGM: 20 | CSII only: 1  CSII + CGM: 1 | CSII only: 13  CSII + CGM: 11 | CSII only: 43  CSII + CGM: 54 |
| **^1^Severe hypoglycemic episodes** | 0 | 2 | 0 | 1 | 0 | 0 | 3 |
|  | -- | CSII only: 0  CSII + CGM: 2 | -- | CSII only: 0  CSII + CGM: 1 | -- | -- | CSII only: 0  CSII + CGM: 3 |
| **^2^Diabetic ketoacidosis** | 4 | 0 | 0 | 0 | 0 | 0 | 4 |
|  | CSII only: 1  CSII + CGM: 3 | CSII only: 0  CSII + CGM: 0 | CSII only: 0  CSII + CGM: 0 | CSII only: 0  CSII + CGM: 0 | CSII only: 0  CSII + CGM: 0 | CSII only: 0  CSII + CGM: 0 | CSII only: 1  CSII + CGM: 3 |
| **Enrolled during pregnancy** | | | | | | | |
| **Number of adverse events** | -- | 24 | 38 | 114 | 2 | 98 | 276 |
|  |  | CSII only: 16  CSII + CGM: 8 | CSII only: 22  CSII + CGM: 16 | CSII only: 55  CSII + CGM: 59 | CSII only: 1  CSII + CGM: 1 | CSII only: 44  CSII + CGM: 54 | CSII only: 138  CSII + CGM: 138 |
| **Death** | -- | 0 | 0 | 0 | 1 | 0 | 1 |
|  |  | -- | CSII only: 1  CSII + CGM: 0 | -- | -- | -- | CSII only: 1  CSII + CGM: 0 |
| **Device-related adverse events** | -- | 0 | 3 | 1 | 0 | 0 | 4 |
|  |  | -- | CSII only: 2  CSII + CGM: 1 | CSII only: 1  CSII + CGM: 0 | -- | -- | CSII only: 3  CSII + CGM: 1 |
| **Serious adverse events** | -- | 23 | 38 | 111 | 2 | 93 | 267 |
|  |  | CSII only: 16  CSII + CGM: 7 | CSII only: 22  CSII + CGM: 16 | CSII only: 55  CSII + CGM: 56 | CSII only: 1  CSII + CGM: 1 | CSII only: 42  CSII + CGM: 51 | CSII only: 136  CSII + CGM: 131 |
| **^1^Severe hypoglycemic episodes** | -- | 4 | 1 | 0 | 0 | 0 | 5 |
|  |  | CSII only: 0  CSII + CGM: 4 | CSII only: 0  CSII + CGM: 1 | -- | --- | -- | CSII only: 0  CSII + CGM: 5 |
| **^2^Diabetic ketoacidosis** | -- | 0 | 1 | 1 | 0 | 0 | 2 |
|  |  | -- | CSII only: 0  CSII + CGM: 1 | CSII only: 1  CSII + CGM: 0 | - | -- | CSII only: 1  CSII + CGM: 1 |

^1^An event requiring the assistance from another person to actively administer carbohydrate, glucagon, or other resuscitative actions.

^2^Blood glucose >250 mg/dL or <13.9 mmol/L) with either low serum bicarbonate (<15 mEq/L) and/or low pH (≤7.24), anion gap (>12), either ketonemia or ketonuria, and requiring treatment within a health-care facility.

T1, T2, T3=Trimester 1, 2, and 3, respectively.

CSII=Continuous subcutaneous insulin infusion.

CGM=Continuous glucose monitoring.
